# Retinal antigen-specific regulatory T cells protect against spontaneous and induced autoimmunity and require local dendritic cells

**DOI:** 10.1186/s12974-014-0205-4

**Published:** 2014-12-11

**Authors:** Scott W McPherson, Neal D Heuss, Mark J Pierson, Dale S Gregerson

**Affiliations:** Department of Ophthalmology and Visual Neurosciences, University of Minnesota, Rm. 310, Lion’s Research Bldg.,2001 6th St. SE., Minneapolis, Minnesota 55455-3007 USA

**Keywords:** regulatory T cells, peripheral regulatory T cells, autoimmunity, dendritic cells

## Abstract

**Background:**

We previously reported that the peripheral regulatory T cells (pTregs) generated ‘on-demand’ in the retina were crucial to retinal immune privilege, and *in vitro* analysis of retinal dendritic cells (DC) showed they possessed antigen presenting cell (APC) activity that promoted development of the Tregs and effector T cells (Teffs). Here, we expanded these findings by examining whether locally generated, locally acting pTregs were protective against spontaneous autoimmunity and autoimmunity mediated by interphotoreceptor retinoid-binding protein (IRBP). We also examined the APC capacity of retinal DC *in vivo*.

**Methods:**

Transgenic (Tg) mice expressing diphtheria toxin receptor (DTR) and/or green fluorescent protein (GFP) under control of the endogenous FoxP3 promoter (GFP only in FG mice, GFP and DTR in FDG mice) or the CD11c promoter (GFP and DTR in CDG mice) were used in conjunction with Tg mice expressing beta-galactosidase (βgal) as retinal neo-self antigen and βgal-specific TCR Tg mice (BG2). Retinal T cell responses were assayed by flow cytometry and retinal autoimmune disease assessed by histological examination.

**Results:**

Local depletion of the Tregs enhanced actively induced experimental autoimmune uveoretinitis to the highly expressed retinal self-antigen IRBP in FDG mice and spontaneous autoimmunity in βgal-FDG-BG2 mice, but not in mice lacking autoreactive T cells or their target antigen in the retina. The presence of retinal βgal downregulated the generation of antigen-specific Teffs and pTregs within the retina in response to local βgal challenge. Retinal DC depletion prevented generation of Tregs and Teffs within retina after βgal injection. Microglia remaining after DC depletion did not make up for loss of DC-dependent antigen presentation.

**Conclusions:**

Our results suggest that local retinal Tregs protect against spontaneous organ-specific autoimmunity and that T cell responses within the retina require the presence of local DC.

## Background

While various types of T cells possess regulatory activity [1,2], it is well established in mice that CD4^+^CD25^+^FoxP3^+^ T cells are a dominant regulatory T cell (Treg) and indispensible for immune homeostasis [3,4]. FoxP3^+^ Tregs are broadly categorized by their origin as either thymically-derived Tregs (tTregs) or as peripherally-derived Tregs (pTregs) [5]. Most FoxP3^+^ Tregs are tTregs. Their generation, along with negative selection of strongly autoreactive T cells in the thymus, provides a foundation against autoimmunity. pTregs are thought to assist tTregs in limiting autoimmune inflammation [6]. In addition, pTregs are important in regulating immune responses to external antigens (Ag) encountered in the gut or airway, providing maternal-fetal tolerance [7-9], as well as tolerance to commensal microbiota [10-12].

Analysis of pTregs for their overall contribution to the Treg population, as well as their exact sites of generation and action (within a specific tissue or nearby secondary lymphoid tissue), is a matter of ongoing investigation. Limiting these studies is the lack of unique marker(s) distinguishing tTregs and pTregs. Some reports suggested the transcription factor Helios [13] and neuropilin-1 [14] specifically marked tTregs. Other studies argued that Helios was upregulated in pTregs and activated T cells [15,16] and was not expressed in all tTregs [17]. Likewise, neuropilin-1 might not be a definitive Treg subset marker as pTregs expressed it during inflammation [18], and its expression was influenced by the local cytokine milieu, particularly TGF-β [19], a factor known to be critical for pTreg development. Nonetheless, it is well established that Tregs are generated in secondary lymphoid tissue as a result of interaction between Ag-bearing dendritic cells (DC) and T cells, and exert their regulatory action by limiting the priming of T cells [20]. Tregs are also known to reside and accumulate in non-lymphoid tissue, especially in response to inflammation. However, it has been difficult to determine if these tissue Tregs resulted from immigration of tTreg or were pTregs generated in secondary lymphoid tissue.

The accumulation of Tregs in non-lymphoid tissues also allows for the possibility that they might be generated directly within the tissue from naïve or effector T cells (Teffs). A recent study showed pTreg development dependent on resident lung macrophages [21]. Although the macrophages were isolated, Ag-pulsed, and then reintroduced in recipient mice by intra-tracheal transfer along with FoxP3^−^ T cells, the resulting pTregs and macrophages were primarily found in lung tissue and not in the mediastinal lymph nodes. The retina, because of its apparent lack of lymphatic drainage [22] and high concentration of TGF-β [23] and retinoic acid [24], might also be a site for tissue-specific pTreg generation. Indeed, injection of naïve T cells specific for the retinal Ag interphotoreceptor retinoid binding protein (IRBP) into the posterior segment of the eye resulted in their conversion to FoxP3^+^ Tregs [25]. Subsequently, we expanded on this result demonstrating that FoxP3^+^ Tregs specific for retinal Ag were generated within the retina from circulating Ag-specific FoxP3^−^ T cells [26]. Further, these pTregs provided a local, specific protection against experimental autoimmune uveoretinitis (EAU) induced by Ag immunization or adoptive transfer of activated, retinal Ag-specific T cells.

Given that central tolerance (negative selection and tTreg generation) to self-Ags is not always complete [27], our results implied that local, ‘on demand’ generation and action of pTregs for tissue specific self-Ags provided a crucial secondary mechanism for immune homeostasis that functioned in immune privileged tissues. Critical to the development and activation of pTregs is interaction with appropriate antigen presenting cells (APC). Microglia (MG) have long been considered to be the primary APC of CNS tissue [28-33]. In quiescent CNS, particularly the retina, there is controversy about the existence, function, and significance of DC as APC [34-37]. However, there is a growing body of evidence for DC in the CNS [38]. We recently demonstrated the presence of DC in quiescent retina, their expansion in response to neural injury, and their capability as APC *in vitro* to generate Teffs and Tregs [39,40].

In this study, we investigated the role of locally generated, locally acting pTregs in immune homeostasis and the ability of retinal DC to support T cell activation and expansion within the retina. Using mice that express *E. coli* beta-galactosidase (βgal mice) as a retinal neo-self Ag along with CD4^+^ T cell receptor transgenic (TCR-Tg) mice specific for βgal, and mice with selectively depletable Tregs or DC, we found that local depletion of Tregs from the retina was sufficient to permit development of spontaneous EAU and that local DC, not MG, were necessary to generate Ag-specific T cell responses within the retina that included pTreg generation.

## Materials and methods

### Mice

The βgal mice (B6-arrβgal mice, MHC haplotype I-A^b^ or B10.A-arrβgal, MHC haplotype I-A^k^) have been described in detail elsewhere [26,41-43]. Briefly, rod photoreceptor cell expression of βgal mimics that of endogenous arrestin, producing approximately 150 ng βgal/retina and < 0.5 ng βgal/pineal gland. Analysis of tissue for βgal expression was done as described [43] with the following modifications: the 12 μM cryostat sections of OCT-embedded tissue were fixed in PBS with 4% paraformaldehyde and 0.25% gluteraldehyde for 7 min and then incubated with X-gal substrate (5-bromo-4-chloro-3-indolyl β-D-galactoside) for 2 to 24 h. The βgalTCR mice (B10.A) and the BG2 mice (C57BL/6 J) mice carry MHC class II restricted (CD4^+^) T cells that recognize βgal protein, specifically epitopes YVVDEANIETHGMV (βgalTCR) or SVTLPAASHAI (BG2), and have been described elsewhere [44,45]. The FG mice, which express green fluorescent protein (GFP) only under control of the endogenous FoxP3 promoter, and FDG mice, which express diphtheria toxin receptor (DTR) and GFP under control of the endogenous FoxP3 promoter, have been described [4,46]; both are C57BL/6 J. The CDG mice (C57BL/6 J) express a chimeric GFP and DTR under control of a transgenic CD11c promoter [47]. Rag^−/−^ mice (RAG-2^−/−^ mice, stock # 008449) were obtained from breeding stock purchased from Jackson Laboratory (Bar Harbor, ME, USA). All mice were negative for the rd8 mutation associated with retinal degeneration [48]. All mice were handled in accordance with the Association for Research in Vision and Ophthalmology (ARVO) Statement for the Use of Animals in Ophthalmic and Vision Research, and the University of Minnesota Institutional Animal Care and Use Committee guidelines. Mice were housed under specific pathogen-free conditions on lactose-free chow.

### Induction and transfer of regulatory T cells

For induction of pTregs in response to soluble Ag, FG mice were injected intravenously (i.v.) with 100 μg of βgal or bovine serum albumin (BSA) solubilized in phosphate buffered saline (PBS) at 1 mg/mL. At 8 days post-injection, CD4^+^ T cells were isolated from pooled lymph nodes (LN) and spleens by magnetic separation (Miltenyi, San Diego, CA, USA) and then sorted by fluorescence-activated cell sorting (FACS) for GFP^+^ (FoxP3^+^ Tregs) cells. The cells were washed and resuspended in PBS to 5 × 10^6^/mL. For induction of pTregs in response to retinal βgal, magnetically purified CD4^+^ T cells from normal FG-BG2 double transgenic mice were sorted by FACS for GFP^−^ cells. The cells were washed and resuspended to 5 × 10^5^/mL. Cells were transferred i.v*.* with recipient mice and number of cells transferred indicated.

### Injections

Diphtheria toxin (DTx), βgal, and saline injections into the eye were done by trans-corneal deposition into the anterior chamber (AC) as previously described [40]. One microliter doses containing saline or the indicated amount of DTx or βgal were given. Systemic depletion of Tregs or DC was done by intraperitoneal (i.p*.*) injections of DTx with dose and timing indicated.

### Analysis of the delayed-type hypersensitive response and enucleations

Analysis of the delayed-type hypersensitive (DTH) response (ear swelling assay) was done by injection of βgal (50 μg in 10 μL) into the ear pinna as previously described [49]. Enucleations were done as previously described [50].

### Induction and analysis of autoimmune disease

EAU was induced by subcutaneous (s.c.) immunization of mice with a single 200-μL dose containing a total of 200 μg of mouse IRBP peptides 1 to 20 (kindly provided in part by Dr. R. Caspi) and 461 to 480 (100 μg each peptide) emulsified in complete Freund’s adjuvant (CFA) containing 5 mg/mL *Mycobacterium tuberculosis* (H37Ra, Sigma, St. Louis, MO, USA) followed by 0.5 μg pertussis toxin (Sigma) per mouse given in 100 μL saline i.p*.* At 21 days post-immunization, the eyes were harvested, fixed in 10% buffered formalin, paraffin embedded, sectioned (5 μM), and stained with hematoxylin and eosin. The slides were examined in a masked fashion and the induced EAU was scored from 0 (no disease) to 5 (complete loss of photoreceptor cells plus damage to the inner layers of the retina) based on histopathological changes in the retina [51].

### Flow cytometry

Pooled spleen and LN cell suspensions from the indicated mice were prepared by tissue homogenization followed by filtration through a 70 μM cell strainer. Lymphocytes were also prepared from whole blood. Red blood cells were lysed using 0.17 M NH_4_Cl, and the remaining cells were washed twice in PBS with the final suspension made in FACS buffer (PBS with 2% FCS and 0.02% sodium azide). 0.25 to 2.0 μL/10^6^ cells of the appropriate fluorescent-labeled antibodies (BD Biosciences, San Jose, CA, USA or eBioscience, San Diego, CA, USA) were added to the cell suspension and incubated on ice for 30 min. The cells were washed, resuspended in FACS buffer, and analyzed on FACSCalibur or FACSCanto flow cytometers using CellQuest (BD Biosciences) or FlowJo (Tree Star, Ashland, OR, USA) software. CD4^+^ T cells from immunized FG mice and normal BG2 x FG mice were sorted into regulatory (GFP^+^) or effector (GFP^−^) populations using a FACSAria flow cytometer (BD Bioscience). For analysis of retinal cells by flow cytometry, mice were euthanized, perfused, and the retinas removed as described [40]. Control experiments showed that the perfusion procedure effectively removed passenger cells from the retinal vasculature so that their contribution was not significant (data not shown). The retinas were dissociated using a solution of 0.2 μg/mL Liberase/TM (Roche, Indianapolis, IN, USA) and 0.05% DNase in PBS, and stained with the indicated antibodies. Gating strategy and analysis of retinal mononuclear cells and lymphocytes has been described [26,40]. For the purpose of analysis, a single sample comprised all cells collected from a single retina.

## Results

### Beta-galactosidase (βgal)-specific regulatory T cells are made in the periphery and modulate CD4^+^ T cell responses to βgal

**Figure 1:**
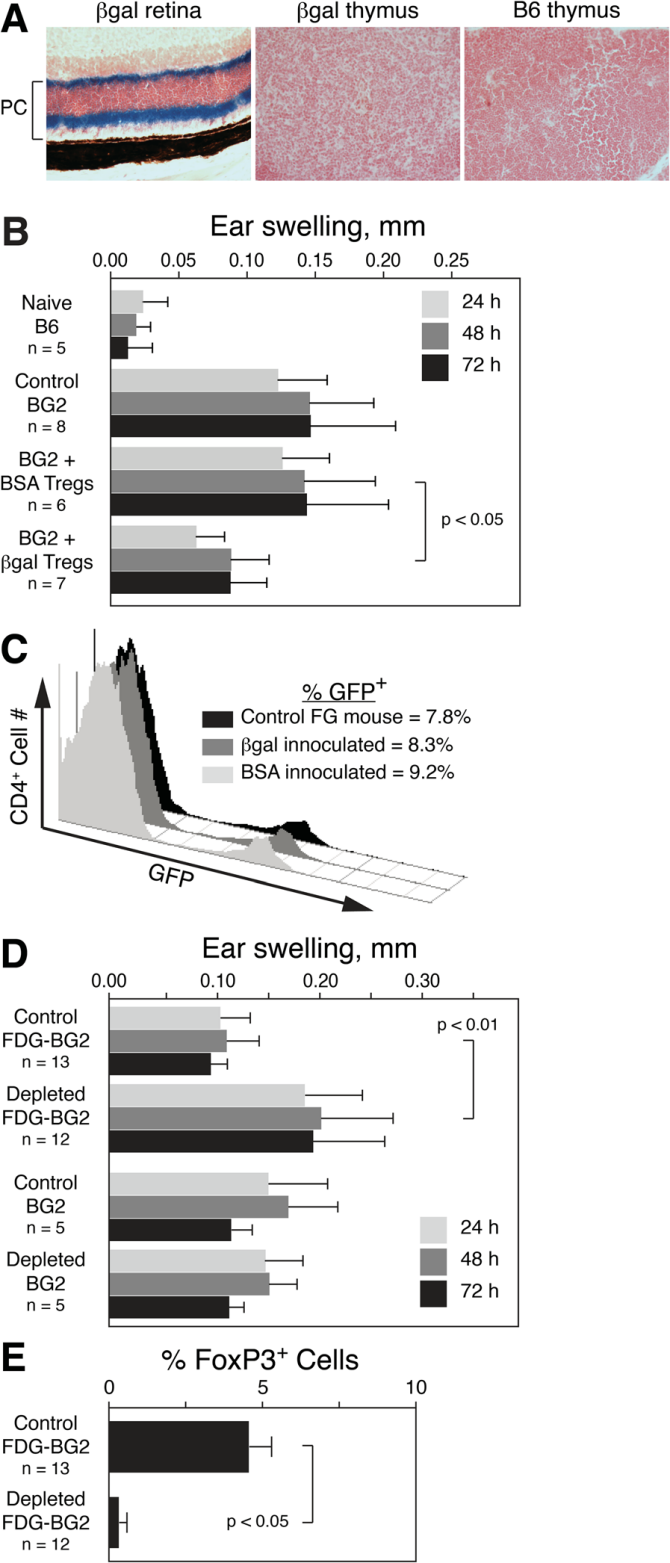
**Regulatory T cells (Tregs) specific for beta-galactosidase (βgal) were generated in the periphery and modulated a CD4** ^**+**^ **T cell mediated delayed-type hypersensitivity (DTH) response. (A)** X-gal staining of retina and thymus from a βgal mouse and control B6 mouse thymus. Photoreceptor (PC) layer of βgal mouse retina indicated showing intense X-gal staining in the outer plexiform layer (top) and outer segments (bottom). Retinas were incubated for 2 h and thymus for 24 h with X-gal. **(B)** Inhibition of CD4^+^ T cell (BG2)-induced DTH to βgal in mice receiving βgal specific Tregs. Transferred mice received 5 x 10^5^ Tregs intravenously (i.v.) from mice injected with bovine serum albumin (BSA) or βgal. Recipient mice were given βgal in the ear and then measured for ear swelling at the indicated times post-βgal injection. **(C)** Percent of pooled CD4^+^ T cells that are Tregs (GFP^+^) in control and antigen (Ag)-inoculated FG mice. **(D)** Enhancement of BG2-induced DTH following systemic depletion of Tregs. Mice were depleted by 250 ng DTx given intraperitoneally (i.p.) on days 0, 3, and 6, followed by βgal in the ear on day 7. Ear swelling was measured at indicated times post-βgal injection. **(E)** Analysis of Treg levels in the blood of control mice and FDG-BG2 mice given DTx i.p*.* at the time of βgal injection in the ear (day 7). Results are given as mean ± SD with *P* values determined by *t* test.

The lack of βgal expression in the thymus of βgal mice by reverse transcription PCR (RT-PCR) [50] and X-gal staining (Figure 1A) suggested that most βgal-specific Tregs are likely pTregs. This was supported by our studies that showed that injection of Ag into the naïve mouse eye led to local generation of pTregs that inhibited EAU [26]. In further support, we sought additional direct evidence for the peripheral generation of βgal-specific Tregs, evidence for their function both in the retina and systemically, and evidence that the presence of the retina affected the generation of circulating pTregs.

To demonstrate that functional, βgal-specific, FoxP3^+^ pTregs could be generated *in vivo*, we asked if Tregs induced by administration of soluble Ag [52] and then transferred into BG2 mice could suppress the DTH response of βgal-specific CD4^+^ BG2 T cells. FG mice were injected i.v. with βgal or BSA. After 8 days, GFP^+^ Tregs from the βgal and BSA treated mice were purified by FACS and transferred into separate groups of naïve BG2 mice. The recipient BG2 mice were then analyzed for their DTH response to βgal. Control BG2 mice had a significant DTH response to βgal compared to naïve B6 mice (Figure 1B). After transfer, BG2 mice that received Tregs from BSA treated mice had no reduction in ear swelling compared to control mice, while mice receiving Tregs from βgal treated mice showed a significant reduction in ear swelling (Figure 1B). FACS analysis showed that the size of the overall Treg population measured as a percent of circulating CD4^+^ T cells was unaffected by Ag injection (Figure 1C), a finding not unexpected given that homeostatic mechanisms tightly regulate the overall size of the T cell pool and its subsets, including Tregs [53-56]. However, the results indicated that Ag-specific, βgal-induced pTregs were generated in sufficient numbers to have a significant effect on the DTH response of βgal-specific CD4^+^ BG2 T cells. Conversely, we also examined whether removal of Tregs would enhance the DTH response to βgal. FDG-BG2 double Tg mice were depleted of Tregs by systemic injections of DTx and then assayed for their DTH response to βgal. Treg depleted FDG-BG2 mice had significantly increased ear swelling compared to control FDG-BG2 mice (Figure 1D, top). To show that the increase ear swelling was specifically due to Treg depletion, we also compared the DTH response in DTx and non-DTx treated BG2 mice (Figure 1D, bottom) and found it was similar to each other and to the undepleted FDG-BG2 mice. Analysis of blood for circulating Tregs showed that DTx treated FDG-BG2 mice were substantially depleted of Tregs compared to control FDG-BG2 mice (Figure 1E). Although the mice in these experiments do not carry the βgal transgene, the results show a detectable level of control of the BG2-mediated DTH response by spontaneously generated Tregs, and that addition (Figure 1B) or depletion (Figure 1D) of even a small number of circulating, βgal-specific Tregs can modulate the immune response to βgal in a site not thought to be immune privileged.

**Figure 2:**
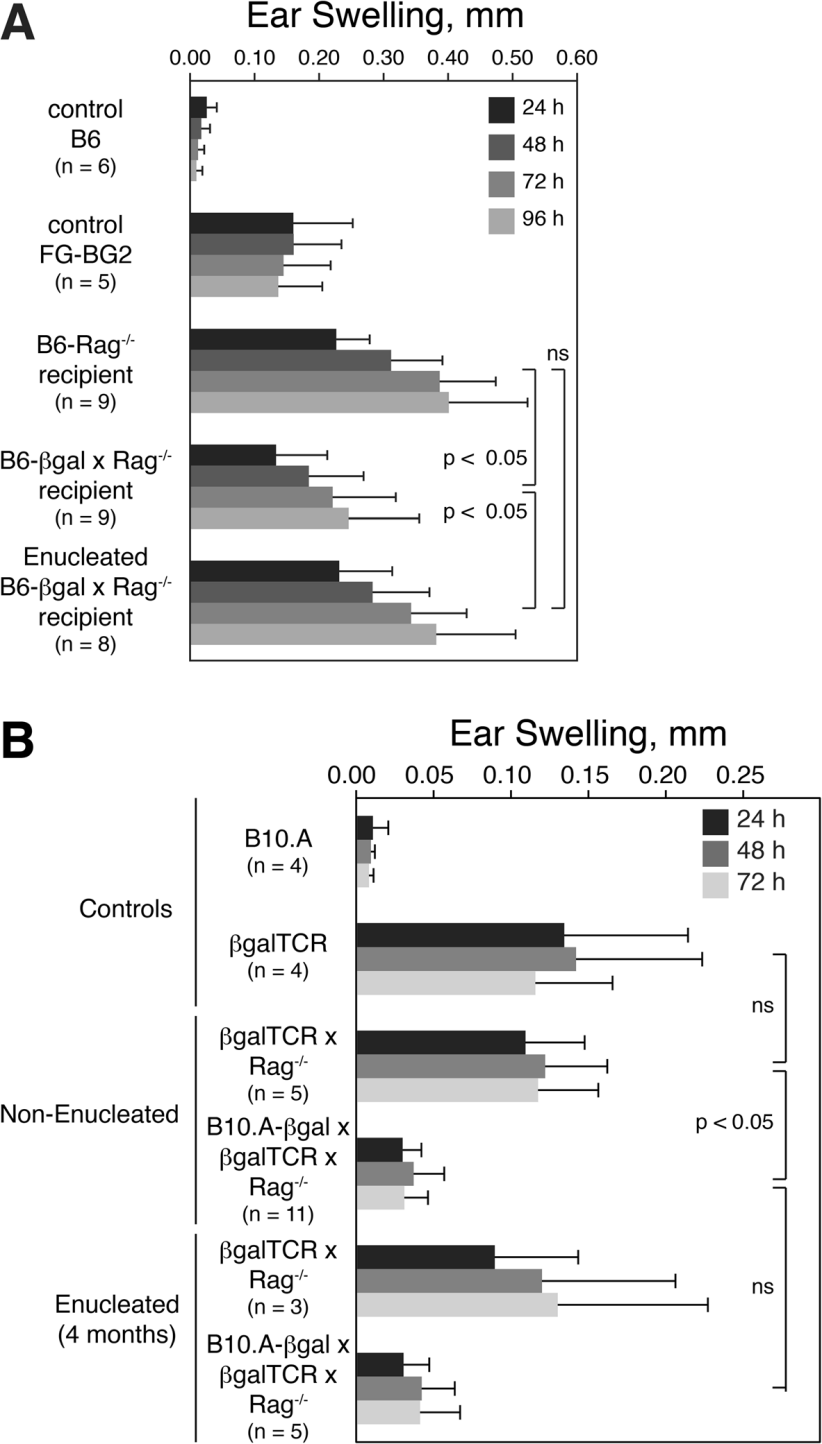
**New, peripherally generated beta-galactosidase** (**βgal)-specific regulatory T cells (Tregs) inhibit delayed-type hypersensitivity (DTH). (A)** Ear swelling assay showing that retinal βgal is required to generate βgal-specific Tregs that can modulate the DTH response to βgal. Mice were analyzed 80 days post-transfer of 5 x 10^4^ CD4^+^GFP^−^ cells from FG-BG2 mice. **(B)** Ear swelling assay showing that the presence of the retina is not necessary for maintaining βgal-specific Tregs. Mice were enucleated and compared to non-enucleated mice 4 months post-enucleation. Ear swelling measured at indicated time post-βgal injection. Results are given as mean ± SD with *P* values determined by *t* test.

Since greater than 99.8% of the βgal in βgal mice is expressed in the retina, a comparison of mice with or without the principal βgal source removed by enucleation would test the role of the retinal Ag in βgal-specific pTreg development. Mature, naïve CD4^+^ T cells from FG-BG2 double Tg mice were sorted by flow cytometry for GFP^−^ (FoxP3^−^) cells and transferred into normal or enucleated B6-βgal x Rag^−/−^ mice. Since Rag^−/−^ mice lack T cells, including endogenous Tregs, all new Tregs must be pTregs derived from the transferred FoxP3^−^ T cells. When challenged with Ag to determine specificity, recipient mice totally lacking βgal (Rag^−/−^ mice) or lacking retinal βgal (enucleated B6-βgal x Rag^−/−^ mice) had an equivalent elevated DTH response compared to βgal^+^ recipients (Figure 2A), suggesting that the retinal βgal promotes the generation of functional retinal Ag-specific pTregs from naïve, mature precursor T cells in the periphery. To assess the stability of retina-dependent pTregs, DTH responses were also analyzed in B10.A-βgal x Rag^−/−^ mice where precursor T cells were provided in the mice by their also having a βgalTCR transgene. At 4 months post-enucleation, we observed similar levels of DTH inhibition in both normal and enucleated βgalTCR x B10.A-βgal x Rag^−/−^ mice compared to similarly treated βgalTCR x Rag^−/−^ mice (Figure 2B). Together, these results show retinal βgal was the primary Ag source for βgal-specific pTreg formation and that once formed, functional βgal-specific Tregs persisted in circulation even if the source of Ag was removed.

### Local regulatory T cell depletion from the retina induces spontaneous experimental autoimmune uveoretinitis

**Figure 3:**
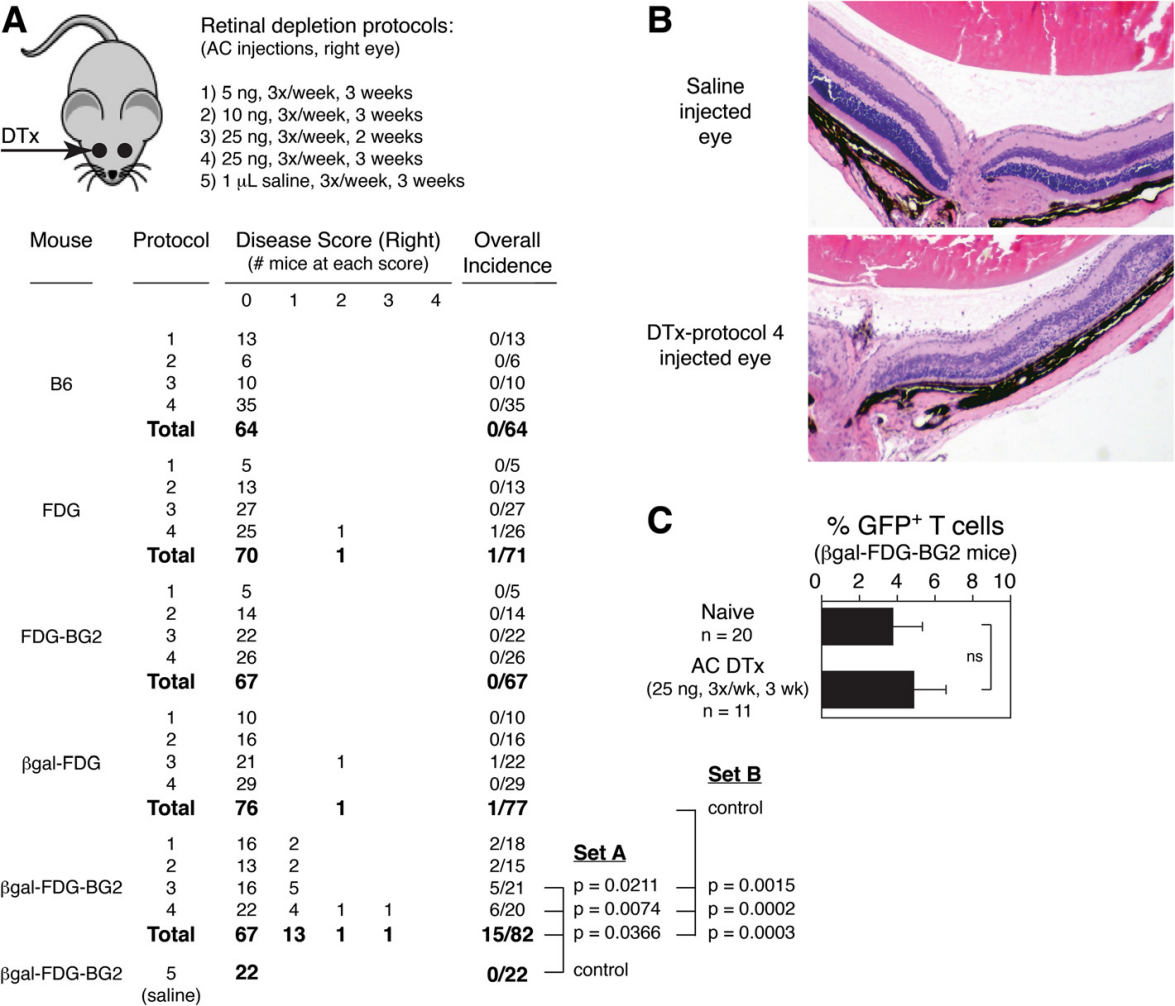
**Local depletion of regulatory T cells (Tregs) induces spontaneous autoimmune disease. (A)** Incidence and severity of experimental autoimmune uveoretinitis (EAU) in the eyes of mice following anterior chamber of the eye (AC) injections of diphtheria toxin (DTx) or saline. Amount, timing, and duration of AC injection protocols are indicated. Statistical set A compares βgal-FDG-BG2 mice treated with saline or DTx, *P* values determined by Fisher’s exact test. Statistical set B compares mice with or without BG2 T cells following DTx treatment, *P* values determined by Fisher’s exact test. **(B)** Representative histology of eyes from βgal-FDG-BG2 mice that received DTx or saline into the right AC. **(C)** Fluorescence-activated cell sorting (FACS) analysis of peripheral blood mononuclear cells showing AC injection of DTx does not alter circulating Treg levels. Results are given as mean ± SD with *P* values determined by *t* test.

B6 mice are minimally permissive for experimental autoimmune uveoretinitis (EAU) [57,58]. Recently, we demonstrated that cells expressing DTR could be locally eliminated from the retina by AC injections of DTx [39,40] and that depletion of retinal Tregs in B6-βgal mice enhanced βgal-mediated EAU induced by adoptive transfer or immunization [26]. To confirm the importance of local Tregs within the retina, we sought evidence that they were also protective against spontaneous autoimmunity. Control B6 mice and strains expressing DTR in FoxP3^+^ cells were given various regimens of DTx into the right AC and analyzed after 3 weeks of treatments (Figure 3A). Control B6 mice given AC DTx did not exhibit any signs of retinal autoimmunity. FDG and βgal-FDG mice each exhibited a rare incident of retinal autoimmune disease (1/71 and 1/77, respectively) following AC depletion of Tregs (Figure 3A). Given that FDG and βgal-FDG mice highly express a number of known, endogenous, immunopathogenic, retinal auto-antigens (for example, IRBP, opsin, arrestin, and recoverin), but do not have an elevated frequency of one particular retinal-Ag specific Teff population, the resulting T cell-mediated autoimmunity could be directed against any of several retinal self-Ags. As expected, FDG-BG2 mice did not develop retinal autoimmunity, as their Teffs are largely βgal-specific thus having only a limited repertoire against self-Ags [26]. However, mice having a high frequency of Teffs specific for βgal plus cognate βgal Ag expression in the retina (βgal-FDG-BG2 mice) did develop EAU at a significant rate (15/82, 18.3%) following retinal Treg depletion, with a general trend of increased incidence with higher DTx doses (Figure 3A). The left eyes of most of the βgal-FDG-BG2 mice were also examined and found to be negative for EAU (0/74, data not shown). Further, βgal-FDG-BG2 mice that were given AC saline injections did not develop retinal autoimmunity in that eye (0/22, Figure 3A, bottom). The lack of disease in the left eyes of DTx treated mice and in the saline injected eyes demonstrates that the autoimmunity in DTx treated eyes was specifically due to the local depletion of Tregs from the retina and that Tregs need to be present within the retina to be protective. Analysis of circulating Tregs showed no difference between naïve and AC DTx treated βgal-FDG-BG2 mice (Figure 3C), again supporting the idea that circulating Tregs play a secondary role in protection of the retina from autoimmunity relative to the contribution of local Tregs.

### Prolonged, systemic regulatory T cell depletion in βgal-FDG-BG2 mice induces spontaneous experimental autoimmune uveoretinitis

**Figure 4:**
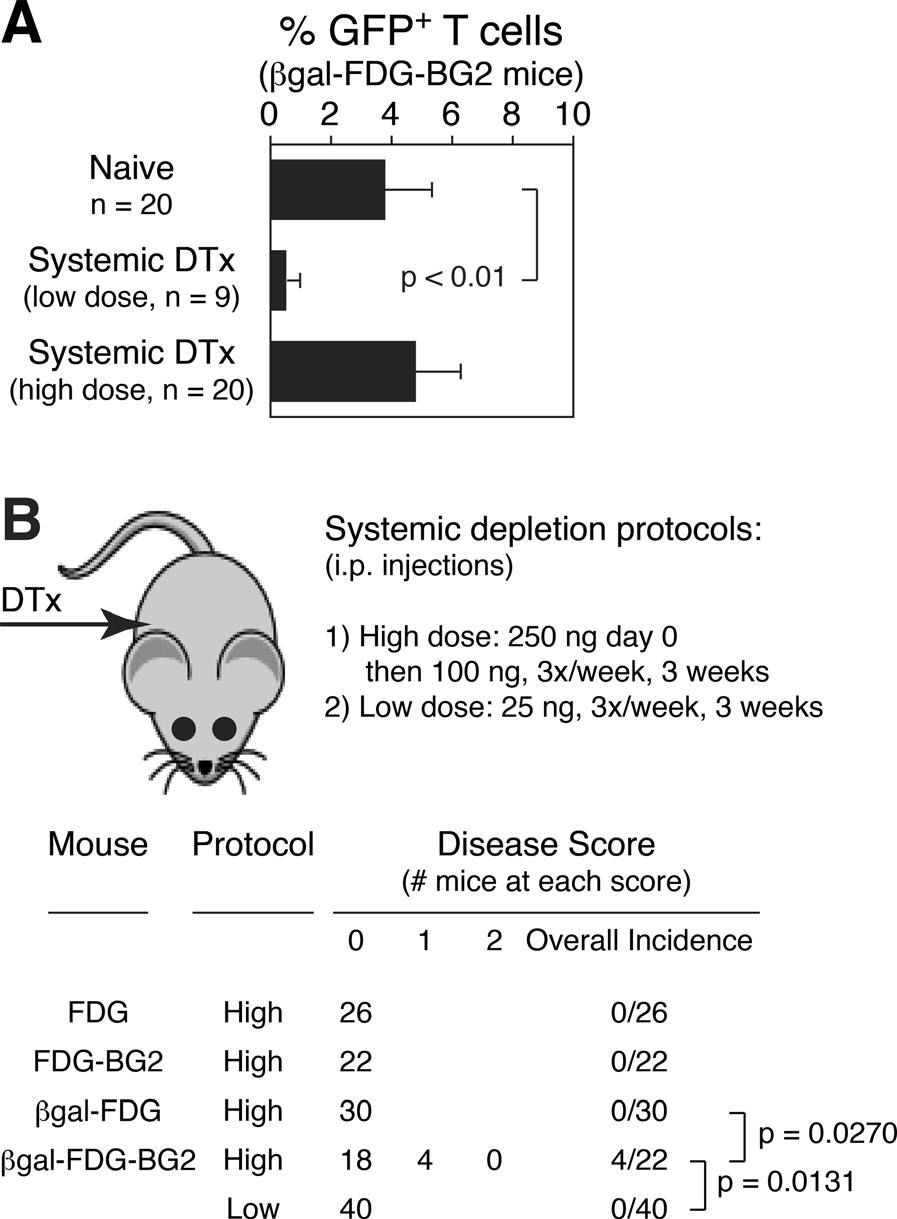
**Systemic depletion of regulatory T cell (Tregs) induces spontaneous autoimmune disease in the retina. (A)** Analysis of circulating Tregs levels following low and high dose systemic depletion protocols. Results are given as mean ± SD with *P* values determined by *t* test. **(B)** Incidence and severity of experimental autoimmune uveoretinitis (EAU) following systemic injections of diphtheria toxin (DTx). Amount, timing, and duration of systemic DTx injection protocols are indicated. *P* values determined by Fisher’s exact test. Analysis of EAU and Treg levels were done at 21 days post-initial DTx injection.

Although DTx given systemically can penetrate the retina and deplete DTR^+^ cells from the retina [40], it was of interest to observe in our previous studies [26] that EAU induced by βgal immunization or adoptive transfer of activated, βgal-specific T cells could not be enhanced by systemic DTx treatment. Since immunization and adoptive transfer protocols result in only a brief window for a limited number of activated T cells to elicit EAU, we hypothesized that systemic DTx treatment of βgal-FDG-BG2 mice might generate EAU at a high rate since the retinas in these mice would be exposed to a very high number of Ag-specific Teffs, as depletion of Tregs would lead to polyclonal T cell activation. The highest dose of AC DTx (25 ng, 3x per week, 3 weeks) when delivered systemically (systemic low dose) did not alter circulating Treg levels compared to naïve mice (Figure 4A) and failed to induce EAU (0/40, Figure 4B), again supporting the importance of local Tregs against autoimmunity. While it is known that systemic Treg depletion results in polyclonal activation of autoreactive T cells leading to progressive multi-organ autoimmune disease [4], a high dose of systemic DTx that resulted in near total Treg depletion (Figure 4A) only induced EAU in βgal-FDG-BG2 mice at a rate similar to the local depletion (4/22, 18.2%, Figure 4B), and not at all in the other strains lacking the combination of βgal-specific TCR-Tg T cells, retinal βgal, and DTx-depletable Tregs (Figure 4B).

### Retinal dendritic cells are necessary for retinal T cell responses

**Figure 5:**
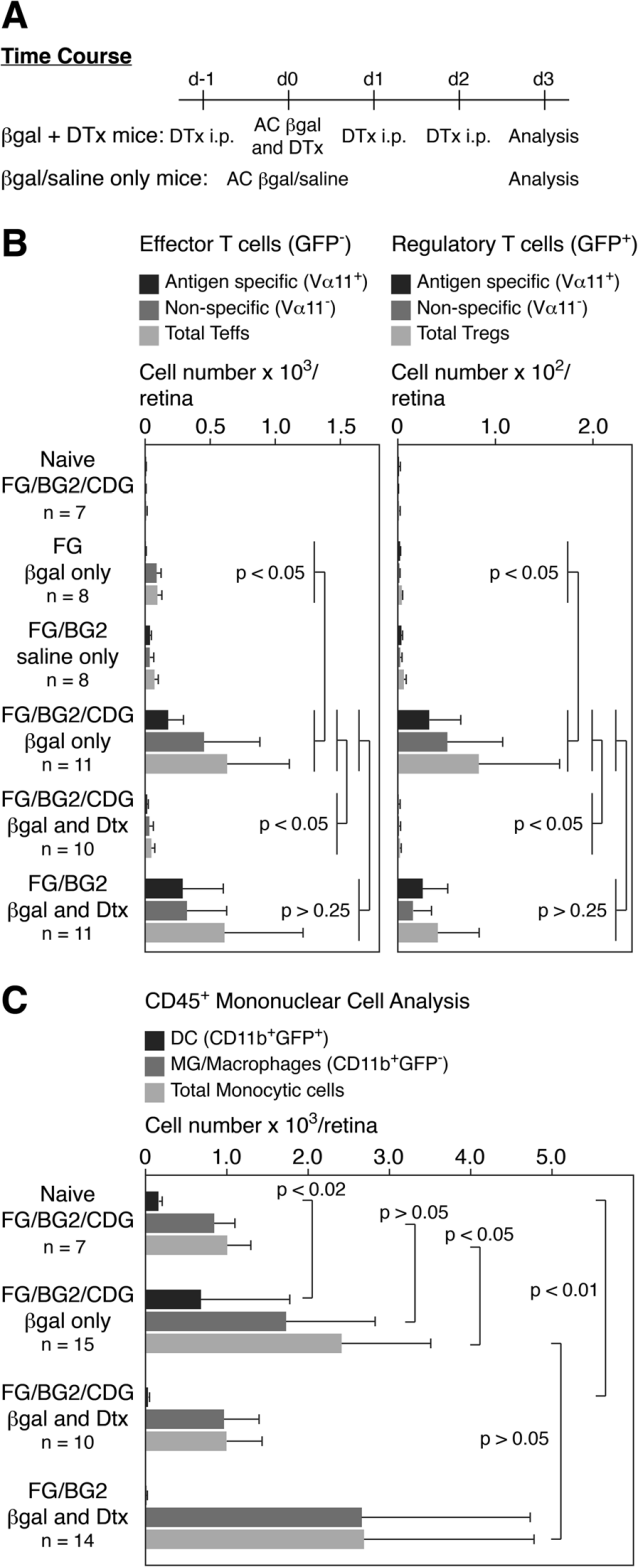
**Depletion of retinal dendritic cell (DC) eliminates the local immune response. (A)** Schematic showing timing and location of diphtheria toxin (DTx) (25 ng), beta-galactosidase (βgal) (10 μg), and saline injections. **(B)** Fluorescence-activated cell sorting (FACS) analysis showing reduction in T cell numbers in retinas depleted of DC. T cells were analyzed as being effector (GFP^−^) or regulatory (GFP^+^) and βgal-specific (Vα11^+^) or nonspecific (Vα11^−^). **(C)** FACS analysis of retinal mononuclear cells showing that DC but not microglia (MG) were reduced in mice treated with DTx. Results are given as mean ± SD with *P* values determined by *t* test.

Small numbers of DC in the quiescent retina can be identified by GFP expression in CDG mice and *in vitro* these retinal DC act as APC [39,40]. To assess whether retinal DC function as APC locally within the retina, we crossed FG-BG2 mice with DC-depletable CDG mice (FG-BG2-CDG mice). While large doses of DTx (200 ng) are highly effective in short-term assays [59], they are eventually lethal to CDG mice. We found that GFP^+^ DC can be depleted from the retina by serial i.p. administration of small doses of DTx (25 ng) for up to 10 days [40]. This, combined with our other finding that AC injection of βgal induces a T cell response within the retina of FG-BG2 mice [26] provided a way to examine the role of retinal DC in the retinal T cell response (Figure 5A). Although in FG-BG2-CDG mice both Tregs and DC are GFP^+^, we distinguished the cells by CD4 and CD11b staining. βgal-specific BG2 T cells are also positive for TCR-Vα11.

Injection of βgal into the AC of FG-BG2-CDG mice stimulated a retinal T cell response that included BG2 (Vα11^+^) and nonspecific Teffs (Vα11^−^), as well as Tregs that were BG2 or nonspecific (Figure 5B). In contrast, non-TCR-Tg (BG2^−^) controls (FG mice) did not have a significant T cell response when given AC injections of βgal (Figure 5B), suggesting that the response of Ag nonspecific Vα11^−^ T cells within the retina of BG2 mice is dependent on generating the βgal-specific Vα11^+^ T cell response that produces cytokines and chemokines that can support the recruitment of Ag nonspecific T cells into the retina. If FG-BG2-CDG mice were also treated with DTx to deplete DC, the entire T cell response (Teff and Treg, BG2 and nonspecific) to βgal was eliminated, yielding T cells numbers similar to naïve FG-BG2-CDG mice and FG-BG2 mice given AC saline (Figure 5B). When given βgal and DTx, control FG-BG2 mice lacking depletable DC had a similar T cell response to βgal stimulated FG-BG2-CDG mice.

We also analyzed the retinal mononuclear cell response (CD11b^+^ cells, but not Ly6G^+^ polymorphonuclear granulocytes). FG-BG2-CDG mice given AC βgal had an elevated number of DC (CD11b^+^GFP^+^ cells) compared to naïve controls, but MG numbers were similar (Figure 5C). However, when these mice were also given DTx, DC numbers were reduced to background while MG numbers were unchanged. Total mononuclear cell numbers in βgal/DTx treated control FG-BG2 mice lacking GFP-labeled, depletable DC were similar to βgal treated FG-BG2-CDG mice (Figure 5C). Together, this data suggested that there is a DC-dependency in the retinal Teff and Treg responses to specific Ag, and that neither MG nor GFP^−^ recruited macrophages contributed to the T cell response.

### Beta-galactosidase in the retina primes it toward immunological unresponsiveness

**Figure 6:**
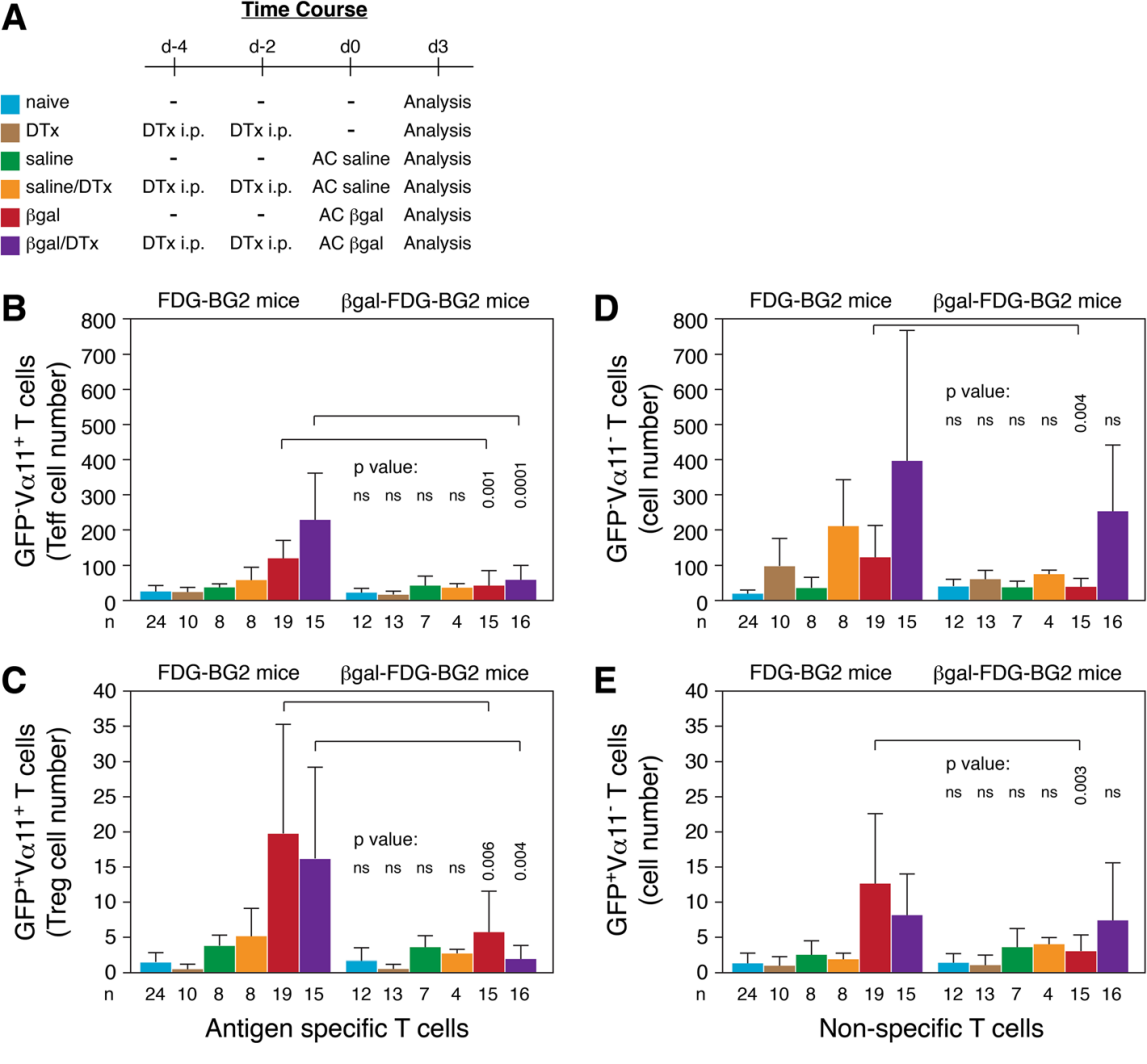
**Retinal beta-galactosidase (βgal) expression induced immunological unresponsiveness. (A)** Legend and time course of the experiments. Mice received 250 ng diphtheria toxin (DTx) and/or βgal (20 μg) or saline as indicated. Retinal T cell analysis was done by fluorescence-activated cell sorting (FACS) 3 days post-injection to the anterior chamber of the eye (AC). **(B-E)** Number of T cells per retina. T cells were analyzed as being effector (GFP^−^) or regulatory (GFP^+^) and βgal-specific (Vα11^+^) or nonspecific (Vα11^−^). Results are given as mean ± SD with *P* values determined by *t* test comparing FDG-BG2 to βgal-FDG-BG2 mice for each type of T cell with significant differences indicated by bracket.

Previously, we established that there is a robust T cell response in the retinas of FDG-BG2 mice to locally administered Ag and that systemic depletion of Tregs did not affect the Ag-stimulated appearance of Tregs within the retina, leading to our conclusion that protective Tregs were made ‘on-demand’ [26]. Given that T cell responses within the retina are dependent on local DC and that DC from quiescent retina favor Treg production [39], we assessed whether the T cell response within the retina would be limited in mice expressing retinal βgal. To ascertain the effect of retinal βgal on the immune response to exogenous Ag, we compared the T cell response in FDG-BG2 and βgal-FDG-BG2 mice following AC injection of βgal with or without systemic pre-depletion of Tregs (Figure 6A). In agreement with our prior findings [26], retinal Teffs (GFP^−^Vα11^+^ and GFP^−^Vα11^−^ cells) increased following AC βgal injection in FDG-BG2 mice, especially with systemic Treg depletion (Figure 6B, D-left side of panels). AC injection of βgal into FDG-BG2 mice also increased retinal Treg numbers (GFP^+^Vα11^+^ and GFP^+^Vα11^−^ cells) regardless of whether they were systemically pre-depleted by treatment with DTx (Figure 6C, E-left side of panels). In contrast, AC βgal injection done in mice expressing retinal βgal resulted in a significantly reduced number of GFP^−^ and GFP^+^ Vα11^+^ T cells (Figure 6B, C-right side compared to left side of panels for βgal) showing that both the Teff and Treg Ag-specific response to βgal was limited. The downregulation of the response was not due to elevated Vα11^+^ Tregs associated with retinal βgal expression as Ag-specific Teffs and Tregs were also reduced between FDG-BG2 and βgal-FDG-BG2 mice following systemic DTx plus AC βgal (Figure 6B, C-right side compared to left side of panels for βgal/DTx). Retinal βgal expression also reduced the Vα11^−^ T cell response to AC βgal injection (Figure 6D, E-right side compared to left side of panels for βgal). However, the number of Vα11^−^ Tregs and Teffs was similar between βgal-FDG-BG2 and FDG-BG2 mice following systemic DTx plus AC βgal (Figure 6D, E-right side compared to left side of panels for βgal/DTx), likely the result of expansion of polyclonal, self-reactive Vα11^−^ T cells no longer under the control of circulating Tregs due to their depletion. These results suggest that βgal expression within the retina creates an Ag-specific, immunologically refractive environment.

### Local depletion of regulatory T cell enhances interphotoreceptor retinoid binding protein-induced experimental autoimmune uveoretinitis

**Figure 7:**
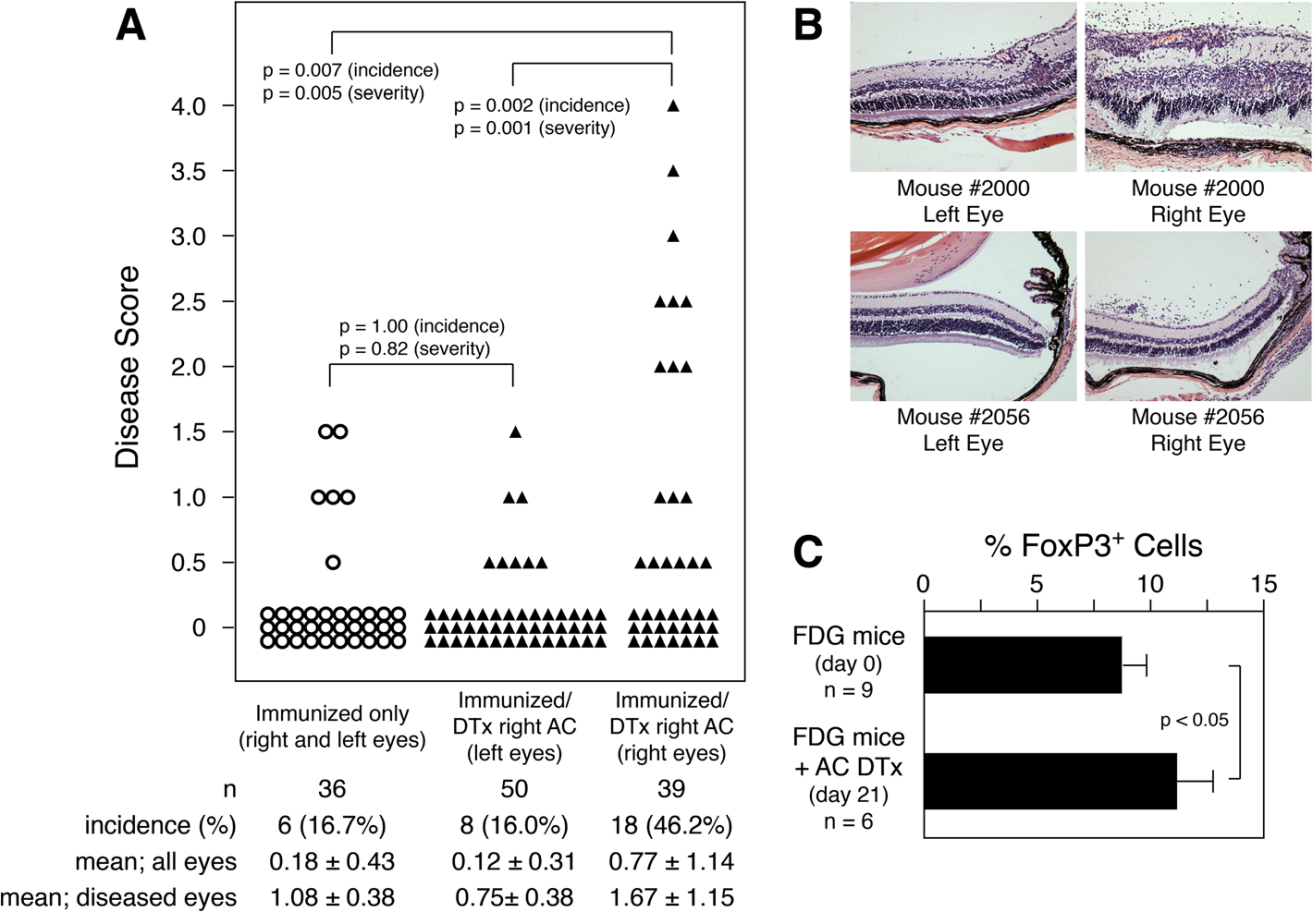
**Interphotoreceptor retinoid binding protein (IRBP)-induced autoimmune disease is enhanced in retinas depleted of regulatory T cells (Tregs). (A)** Incidence and severity of experimental autoimmune uveoretinitis (EAU) in IRBP-immunized only FDG mice and in right and left eyes of FDG mice that were IRBP-immunized and given diphtheria toxin (DTx) into the right anterior chamber of the eye (AC). Mice were given 25 ng of DTx 3 times per week for 3 weeks starting the day of immunization. Retinas were analyzed for EAU at 21 days post-immunization. **(B)** Histology of right and left eyes from IRBP-immunized mice given DTx into the right AC. **(C)** Analysis of blood for Tregs in naïve (day 0) and DTx-treated FDG mice (day 21). For disease scores, mean and SD are given, *P* values were determined by Fisher’s exact test for incidence and Mann–Whitney test for severity. For blood analysis, results are given as mean ± SD with *P* value determined by *t* test.

B6 mice are minimally permissive for EAU [57,58]. Recently, we demonstrated that cells expressing DTR could be locally eliminated from the retina by AC injections of DTx [39,40], and that depletion of retinal Tregs in B6-βgal mice enhanced βgal-mediated EAU [26]. To assess whether our findings extended to an endogenous retinal Ag, we asked if retinal depletion of Tregs could also enhance EAU induced by IRBP, an extracellular retinal protein with a much greater expression level then βgal, and the most common retinal self-Ag used to induce EAU in mice [58]. FDG mice were immunized with peptides of IRBP, with or without injection of DTx into the right AC. Immunized only mice, as well as the left eyes of immunized mice that received AC injections of DTx, developed a similarly modest incidence and severity of EAU (Figure 7A, B). However, Treg-depleted retinas from right eyes had a significant enhancement in the incidence and severity of EAU. Analysis of blood showed that the AC injections of DTx did not lower circulating Treg levels but actually resulted in a slight increase over the course of the experiment (Figure 7C). Given that DTx only eliminates existing Tregs while not preventing new Treg generation, the selective pressure to maintain Tregs, and the low dose of DTx used, it was not surprising to find that circulating Tregs were not lost. Consistent with our previous findings using βgal as a target neo-self Ag, these results suggest that Tregs need to be present within the retina to have a protective effect.

## Discussion

Previously we demonstrated that antigen-specific pTregs could be generated in an ‘on-demand’ manner within the retina and be protective against experimentally induced autoimmunity directed against the neo-self antigen βgal [26], suggesting that pTregs are an important mechanism for homeostasis of immune privileged tissue. In this study we further assessed the nature of retinal pTregs and showed they are protective against autoimmune disease directed at the endogenous retinal antigen IRBP and also protective against spontaneous retinal autoimmunity. We also assessed the role of retinal APC, providing evidence that it is the local DC, and not MG, within the retina that are crucial for generating both Teff and Treg responses to retinal Ags.

Using βgal-specific CD4^+^ TCR Tg (BG2) mice in this study along with βgal-specific CD8^+^ TCR Tg (BG1) mice in our previous study [60], we found that the DTH response to βgal could be modulated by the addition or subtraction of FoxP3^+^ Tregs. Although βgal expression was absent in these TCR Tg mice, the ability to up or down regulate the DTH response by altering Treg levels implies a certain baseline amount of tTreg-mediated control in naïve TCR Tg mice. It has been demonstrated that T cells specific for foreign or neo-self Ags can be positively selected in the thymus via cross-reactive (Ag-mimicking) self-peptides [61,62]. This accounts for the Ag-specific T cell response in wild-type mice following βgal immunization and allows for the formal possibility that naïve mice have a limited number of βgal-reactive tTregs, especially BG2 mice given their large number of Ag-specific precursor T cells. Nonetheless, experiments herein using Rag^−/−^ mice transferred with FoxP3^−^ precursor T cells clearly demonstrated the Ag-dependent peripheral generation of βgal-specific Tregs. Combined with our previous results [26], we propose that locally generated, locally acting, Ag-specific pTregs are a crucial factor in the contribution of Tregs towards retinal immune privilege and that tTregs to βgal generated by selection on cross-reactive self-peptides are limited in number and function.

Underscoring the importance of local Tregs in retinal immune privilege is the observation that local Treg depletion from the retina enhanced IRBP-induced EAU, recapitulating the results found with retinal βgal [26]. In addition to being highly expressed in the retina, there is also *aire* promoted expression of IRBP in the thymus [63,64] providing a certain level of tolerance to IRBP-mediated autoimmunity mediated by negative selection and generation of Ag-specific tTregs. While these studies clearly demonstrated the importance of central tolerance to IRBP in mitigating autoimmunity, there are circumstances that suggest that locally generated IRBP-specific pTregs also contribute to retinal immune privilege. First, based on the amount of IRBP in the human retina [65] and the relative size of the human and murine retina [66-68], we estimate endogenous IRBP in the retina to be at least tenfold the concentration of retinal βgal in the βgal mice. This high level of IRBP expression in an environment that is primed by high concentrations of TGF-β and retinoic acid for Treg production [69-72] makes IRBP-specific pTreg generation likely. Second, an IRBP-specific Teff population that escaped negative selection has been described [73] and thus making obvious a need for peripheral regulation. While it has been shown that this T cell population has limited pathogenicity [73,74], the ability to regulate its priming and effector functions within the retina would be an efficient method of control. Unlike our previous studies with βgal, we did not formally demonstrate herein that IRBP-specific pTregs are made in the retina. However, our results combined with other investigations suggest that it is likely, if not necessary, that there be local production of IRBP-specific pTregs.

Our retinal βgal/BG2 TCR Tg/FoxP3-DTR model system is well suited for demonstrating the role of pTregs in tissue-specific immune privilege in that naive mice, whether expressing one or any combination of the transgenes, do not develop autoimmune disease. This is in contrast to other models of autoimmune disease, including EAU, using TCR Tg mice specific for self-Ag or neo-self Tg Ag that actually develop a high level of spontaneous autoimmune disease [75-77] despite thymic expression of the Ag. In these models, autoimmunity induced by Treg depletion might not be distinguishable from the spontaneous autoimmunity. Since we had demonstrated that retinal depletion of Tregs enhanced EAU in βgal-FDG mice that were either βgal-immunized or transferred with activated βgal-specific T cells [26] it was logical to ascertain whether the same depletion regimen could induce autoimmunity in otherwise naive βgal-FDG-BG2 mice. Our finding that significant EAU was only found in DTx-treated eyes of the triple Tg mice and not in their contralateral eye nor in mice lacking one or more of the transgenes further supports the idea that local action of Ag-specific Tregs is a crucial component of retinal immune privilege.

An interesting difference between immunized/transferred βgal-FDG mice of our previous study [26] and βgal-FDG-BG2 mice herein was that significant systemic Treg depletion induced by high doses of DTx caused EAU only in βgal-FDG-BG2 mice and then only at a rate equal to that observed with local depletion. In rodent strains with limited susceptibility for EAU [57], the highest incidence and severity of disease is associated with situations that involve a chronic stimulation of a large T cell population such as lymphopenia [44], activation by commensal microbiota [76,78,79], antigen mimicry [80], and Treg loss or lack of production [64,81]. Given the high frequency of βgal-specific T cells in βgal-FDG-BG2 mice, it is likely that the lympho-proliferation associated with systemic Treg depletion [4] resulted in a significant T cell activation that was sufficient to overcome the on-going, local production of pTregs in the retina. Although higher doses of DTx or longer treatment would be lethal to βgal-FDG-BG2 mice, we speculate that it would result in higher incidence and severity of EAU. Nonetheless, the results herein and previously [26] suggest that it is the local Tregs within the retina, and their on-going generation, that sets the threshold for retinal autoimmune disease.

Our retinal βgal/BG2 TCR Tg/FoxP3-DTR model system is also useful in examining the initial, critical component of local immune regulation - namely the effect of Ag expression within the tissue on the immune response. Meaningful comparison of retinal T cells from naïve mice that do or do not express retinal βgal is difficult due to their intrinsically low numbers within the retina. However, a direct and local antigenic challenge of BG2 mice revealed that those also expressing retinal βgal were highly resistant to generating a T cell response, even in mice whose Tregs were pre-depleted by systemic injection of DTx. This finding is consistent with our previous observation that DC from naïve retina favor Treg production. DC within βgal expressing naïve retina generate βgal-specific Tregs, which in turn create and maintain an immunologically quiescent local environment. Alternatively, or in conjunction with enhanced Treg production, expression of βgal within the retina could induce T cell anergy. This would most likely occur in quiescent retinas that have few DC. This allows other cells with APC ability, such as MG, to present self-Ag. However, retinal APC other than DC may lack the proper co-stimulatory molecules to activate T cells, a situation well understood to generate T cell anergy [82,83]. Regardless of the mechanisms, the tenacity of this immunological unresponsiveness is evidenced by our observations herein and previously [26] that unilateral treatment generating a high rate of EAU in ipsilateral eyes of βgal-FDG mice and βgal-FDG-BG2 mice fails to generate disease within contralateral eyes.

While the presence of DC and their function as APC within the retina is still a matter of active investigation, recent *in vitro* studies have provided evidence that direct contact between DC and T cells is necessary for retinal T cell responses. In addition to our report showing Teff and Treg generation mediated by purified retinal DC [39], it has also been demonstrated that CD4^+^ T cells specific for an ocular transgene required DC plus Ag to become pathogenic [84]. Further, we also demonstrated in a model of EAU mediated by CD8^+^ T cells, in conjunction with MHC class I^−/−^ mice, that pathogenicity requires only the resident retinal cells be MHC class I^+^ [60], again suggesting local but not recruited APC are crucial for retinal T cell responses. Use of a mouse combining the FG transgene, which identifies Tregs but lacks DTR, with the transgenes of the CDG and BG2 mice allowed us to exam directly *in vivo* if retinal DC were significant contributors to the APC function in retina. Other retinal cells including endothelial cells [85,86], retinal pigment epithelial cells [87,88], and especially MG [28-33] have been proposed to have APC activity. Although these putative retinal APC vastly outnumber retinal DC and are not depleted by DTx treatment in mice with the CDG transgene, we found that generation of Teffs and Tregs within the retina as well as T cell mediated pathogenicity was completely dependent on DTR^+^GFP^+^ retinal DC. While these other cell types have been shown to modulate T cell responses and perhaps have secondary APC function, their apparent lack of function as APC in the absence of DC clearly demonstrates that retinal DC are required for initiation of retinal T cell responses.

## Conclusions

In conclusion, we have demonstrated that the retinal environment is capable of Ag-specific pTreg generation and that those pTregs act locally within the retina to limiting both spontaneous and induced autoimmune disease. We have also demonstrated that the presence of βgal within the retina limits the T cell response to challenge with exogenous βgal and that local DC within the retina are critical for generating T cell responses within the retina.

## Abbreviations

AC: anterior chamber of the eye
Ag: antigen
APC: antigen presenting cell
βgal: beta-galactosidase
BSA: bovine serum albumin
CDG: mice expressing a DTR/GFP fusion protein under control of the CD11c promoter
DC: dendritic cell
DTH: delayed-type hypersensitivity
DTR: diphtheria toxin receptor
DTx: diphtheria toxin
EAU: experimental autoimmune uveoretinitis
FACS: fluorescence-activated cell sorting
FG: mice expressing GFP under control of the FoxP3 promoter
FDG: mice expressing DTR and GFP under control of the FoxP3 promoter
GFP: green fluorescence protein
IRBP: interphotoreceptor retinoid binding protein
MG: microglia
pTreg: peripherally derived Treg
Teff: effector T cell
Tg: transgenic
Treg: regulatory T cell
tTreg: thymically derived Treg

## References

[1] Jutel M, Akdis CA (2008). T-cell regulatory mechanisms in specific immunotherapy. Chem Immunol Allergy.

[2] Shevach EM (2006). From vanilla to 28 flavors: multiple varieties of T regulatory cells. Immunity.

[3] Hill JA, Benoist C, Mathis D (2007). Treg cells: guardians for life. Nat Immunol.

[4] Kim JM, Rasmussen JP, Rudensky AY (2007). Regulatory T cells prevent catastrophic autoimmunity throughout the lifespan of mice. Nat Immunol.

[5] Abbas AK, Benoist C, Bluestone JA, Campbell DJ, Ghosh S, Hori S, Jiang S, Kuchroo VK, Mathis D, Roncarolo MG, Rudensky A, Sakaguchi S, Shevach EM, Vignali DA, Ziegler SF (2013). Regulatory T cells: recommendations to simplify the nomenclature. Nat Immunol.

[6] Haribhai D, Williams JB, Jia S, Nickerson D, Schmitt EG, Edwards B, Ziegelbauer J, Yassai M, Li SH, Relland LM, Wise PM, Chen A, Zheng YQ, Simpson PM, Gorski J, Salzman NH, Hessner MJ, Chatila TA, Williams CB (2011). A requisite role for induced regulatory T cells in tolerance based on expanding antigen receptor diversity. Immunity.

[7] Erlebacher A (2013). Mechanisms of T cell tolerance towards the allogeneic fetus. Nat Rev Immunol.

[8] Rowe JH, Ertelt JM, Xin L, Way SS (2012). Pregnancy imprints regulatory memory that sustains anergy to fetal antigen. Nature.

[9] Samstein RM, Josefowicz SZ, Arvey A, Treuting PM, Rudensky AY (2012). Extrathymic generation of regulatory T cells in placental mammals mitigates maternal-fetal conflict. Cell.

[10] de Lafaille MAC, Lafaille JJ (2009). Natural and adaptive foxp3+ regulatory T cells: more of the same or a division of labor?. Immunity.

[11] Josefowicz SZ, Niec RE, Kim HY, Treuting P, Chinen T, Zheng Y, Umetsu DT, Rudensky AY (2012). Extrathymically generated regulatory T cells control mucosal TH2 inflammation. Nature.

[12] de Lafaille MAC, Kutchukhidze N, Shen S, Ding Y, Yee H, Lafaille JJ (2008). Adaptive Foxp3+ regulatory T cell-dependent and -independent control of allergic inflammation. Immunity.

[13] Thornton AM, Korty PE, Tran DQ, Wohlfert EA, Murray PE, Belkaid Y, Shevach EM (2010). Expression of Helios, an Ikaros transcription factor family member, differentiates thymic-derived from peripherally induced Foxp3+ T regulatory cells. J Immunol.

[14] Yadav M, Louvet C, Davini D, Gardner JM, Martinez-Llordella M, Bailey-Bucktrout S, Anthony BA, Sverdrup FM, Head R, Kuster DJ, Ruminski P, Weiss D, Von Schack D, Bluestone JA (2012). Neuropilin-1 distinguishes natural and inducible regulatory T cells among regulatory T cell subsets in vivo. J Exp Med.

[15] Akimova T, Beier UH, Wang L, Levine MH, Hancock WW (2011). Helios expression is a marker of T cell activation and proliferation. PLoS One.

[16] Gottschalk RA, Corse E, Allison JP (2012). Expression of Helios in peripherally induced Foxp3+ regulatory T cells. J Immunol.

[17] Himmel ME, MacDonald KG, Garcia RV, Steiner TS, Levings MK (2013). Helios + and Helios- cells coexist within the natural FOXP3+ T regulatory cell subset in humans. J Immunol.

[18] Yadav M, Stephan S, Bluestone JA (2013). Peripherally induced tregs - role in immune homeostasis and autoimmunity. Front Immunol.

[19] Weiss JM, Bilate AM, Gobert M, Ding Y, de Lafaille MA C, Parkhurst CN, Xiong H, Dolpady J, Frey AB, Ruocco MG, Yang Y, Floess S, Huehn J, Oh S, Li MO, Niec RE, Rudensky AY, Dustin ML, Littman DR, Lafaille JJ (2012). Neuropilin 1 is expressed on thymus-derived natural regulatory T cells, but not mucosa-generated induced Foxp3+ T reg cells. J Exp Med.

[20] DiPaolo RJ, Brinster C, Davidson TS, Andersson J, Glass D, Shevach EM (2007). Autoantigen-specific TGFbeta-induced Foxp3+ regulatory T cells prevent autoimmunity by inhibiting dendritic cells from activating autoreactive T cells. J Immunol.

[21] Soroosh P, Doherty TA, Duan W, Mehta AK, Choi H, Adams YF, Mikulski Z, Khorram N, Rosenthal P, Broide DH, Croft M (2013). Lung-resident tissue macrophages generate Foxp3+ regulatory T cells and promote airway tolerance. J Exp Med.

[22] Xu H, Chen M, Reid DM, Forrester JV (2007). LYVE-1-positive macrophages are present in normal murine eyes. Invest Ophthalmol Vis Sci.

[23] Niederkorn JY (2006). See no evil, hear no evil, do no evil: the lessons of immune privilege. Nat Immunol.

[24] Keino H, Watanabe T, Sato Y, Okada AA (2010). Anti-inflammatory effect of retinoic acid on experimental autoimmune uveoretinitis. Br J Ophthalmol.

[25] Zhou R, Horai R, Silver PB, Mattapallil MJ, Zarate-Blades CR, Chong WP, Chen J, Rigden RC, Villasmil R, Caspi RR (2012). The living eye “disarms” uncommitted autoreactive T cells by converting them to Foxp3(+) regulatory cells following local antigen recognition. J Immunol.

[26] McPherson SW, Heuss ND, Gregerson DS (2013). Local “on-demand” generation and function of antigen-specific Foxp3+ regulatory T cells. J Immunol.

[27] Gallegos AM, Bevan MJ (2006). Central tolerance: good but imperfect. Immunol Rev.

[28] Harms AS, Cao S, Rowse AL, Thome AD, Li X, Mangieri LR, Cron RQ, Shacka JJ, Raman C, Standaert DG (2013). MHCII is required for alpha-synuclein-induced activation of microglia, CD4 T cell proliferation, and dopaminergic neurodegeneration. J Neurosci.

[29] Jaini R, Popescu DC, Flask CA, Macklin WB, Tuohy VK (2013). Myelin antigen load influences antigen presentation and severity of central nervous system autoimmunity. J Neuroimmunol.

[30] Jarry U, Jeannin P, Pineau L, Donnou S, Delneste Y, Couez D (2013). Efficiently stimulated adult microglia cross-prime naive CD8+ T cells injected in the brain. Eur J Immunol.

[31] Scheffel J, Regen T, Van Rossum D, Seifert S, Ribes S, Nau R, Parsa R, Harris RA, Boddeke HW, Chuang HN, Pukrop T, Wessels JT, Jurgens T, Merkler D, Bruck W, Schnaars M, Simons M, Kettenmann H, Hanisch UK (2012). Toll-like receptor activation reveals developmental reorganization and unmasks responder subsets of microglia. Glia.

[32] Almolda B, Gonzalez B, Castellano B (2011). Antigen presentation in EAE: role of microglia, macrophages and dendritic cells. Front Biosci (Landmark Ed).

[33] Steinbach K, Piedavent M, Bauer S, Neumann JT, Friese MA (2013). Neutrophils amplify autoimmune central nervous system infiltrates by maturing local APCs. J Immunol.

[34] Greter M, Heppner FL, Lemos MP, Odermatt BM, Goebels N, Laufer T, Noelle RJ, Becher B (2005). Dendritic cells permit immune invasion of the CNS in an animal model of multiple sclerosis. Nat Med.

[35] McMahon EJ, Bailey SL, Miller SD (2006). CNS dendritic cells: critical participants in CNS inflammation?. Neurochem Int.

[36] Xu H, Chen M, Mayer EJ, Forrester JV, Dick AD (2007). Turnover of resident retinal microglia in the normal adult mouse. Glia.

[37] Xu H, Dawson R, Forrester JV, Liversidge J (2007). Identification of novel dendritic cell populations in normal mouse retina. Invest Ophthalmol Vis Sci.

[38] D’Agostino PM, Gottfried-Blackmore A, Anandasabapathy N, Bulloch K (2012). Brain dendritic cells: biology and pathology. Acta Neuropathol.

[39] Heuss ND, Lehmann U, Norbury CC, McPherson SW, Gregerson DS (2012). Local activation of dendritic cells alters the pathogenesis of autoimmune disease in the retina. J Immunol.

[40] Lehmann U, Heuss ND, McPherson SW, Roehrich H, Gregerson DS (2010). Dendritic cells are early responders to retinal injury. Neurobiol Dis.

[41] Kikuchi T, Raju K, Breitman ML, Shinohara T (1993). The proximal promoter of the mouse arrestin gene directs gene expression in photoreceptor cells and contains an evolutionarily conserved retinal factor-binding site. Mol Cell Biol.

[42] Kimura A, Singh D, Wawrousek EF, Kikuchi M, Nakamura M, Shinohara T (2000). Both PCE-1/RX and OTX/CRX interactions are necessary for photoreceptor- specific gene expression. J Biol Chem.

[43] Gregerson DS, Torseth JW, McPherson SW, Roberts JP, Shinohara T, Zack DJ (1999). Retinal expression of a neo-self antigen, beta-galactosidase, is not tolerogenic and creates a target for autoimmune uveoretinitis. J Immunol.

[44] McPherson SW, Heuss ND, Gregerson DS (2009). Lymphopenia-induced proliferation is a potent activator for CD4+ T cell-mediated autoimmune disease in the retina. J Immunol.

[45] Tewalt EF, Grant JM, Granger EL, Palmer DC, Heuss ND, Gregerson DS, Restifo NP, Norbury CC (2009). Viral sequestration of antigen subverts cross presentation to CD8(+) T cells. PLoS Pathog.

[46] Fontenot JD, Rasmussen JP, Williams LM, Dooley JL, Farr AG, Rudensky AY (2005). Regulatory T cell lineage specification by the forkhead transcription factor foxp3. Immunity.

[47] Jung S, Unutmaz D, Wong P, Sano G, De los Santos K, Sparwasser T, Wu S, Vuthoori S, Ko K, Zavala F, Pamer EG, Littman DR, Lang RA (2002). In vivo depletion of CD11c(+) dendritic cells abrogates priming of CD8(+) T cells by exogenous cell-associated antigens. Immunity.

[48] Mattapallil MJ, Wawrousek EF, Chan CC, Zhao H, Roychoudhury J, Ferguson TA, Caspi RR (2012). The Rd8 mutation of the Crb1 gene is present in vendor lines of C57BL/6 N mice and embryonic stem cells, and confounds ocular induced mutant phenotypes. Invest Ophthalmol Vis Sci.

[49] Gregerson DS, Dou C (2002). Spontaneous induction of immunoregulation by an endogenous retinal protein. Invest Ophthalmol Vis Sci.

[50] Gregerson DS, Heuss ND, Lehmann U, McPherson SW (2009). Peripheral induction of tolerance by retinal antigen expression. J Immunol.

[51] Gregerson DS, Obritsch WF, Donoso LA (1993). Oral tolerance in experimental autoimmune uveoretinitis. Distinct mechanisms of resistance are induced by low dose vs high dose feeding protocols. J Immunol.

[52] Thorstenson KM, Khoruts A (2001). Generation of anergic and potentially immunoregulatory CD25 + CD4 T cells in vivo after induction of peripheral tolerance with intravenous or oral antigen. J Immunol.

[53] Marie JC, Letterio JJ, Gavin M, Rudensky AY (2005). TGF-beta1 maintains suppressor function and Foxp3 expression in CD4 + CD25+ regulatory T cells. J Exp Med.

[54] Setoguchi R, Hori S, Takahashi T, Sakaguchi S (2005). Homeostatic maintenance of natural Foxp3(+) CD25(+) CD4(+) regulatory T cells by interleukin (IL)-2 and induction of autoimmune disease by IL-2 neutralization. J Exp Med.

[55] Simonetta F, Gestermann N, Martinet KZ, Boniotto M, Tissieres P, Seddon B, Bourgeois C (2012). Interleukin-7 influences FOXP3 + CD4+ regulatory T cells peripheral homeostasis. PLoS One.

[56] Tang Q, Henriksen KJ, Boden EK, Tooley AJ, Ye J, Subudhi SK, Zheng XX, Strom TB, Bluestone JA (2003). Cutting edge: CD28 controls peripheral homeostasis of CD4 + CD25+ regulatory T cells. J Immunol.

[57] Egwuagu CE, Charukamnoetkanok P, Gery I (1997). Thymic expression of autoantigens correlates with resistance to autoimmune disease. J Immunol.

[58] Agarwal RK, Silver PB, Caspi RR (2012). Rodent models of experimental autoimmune uveitis. Methods Mol Biol.

[59] Heuss ND, Pierson MJ, Montaniel K, McPherson SW, Lehmann U, Hussong SA, Ferrington DA, Low WC, Gregerson DS (2014). Retinal dendritic cell recruitment, but not function, was inhibited in MyD88 and TRIF deficient mice. J Neuroinflammation.

[60] McPherson SW, Heuss ND, Gregerson DS (2012). Regulation of CD8(+) T cell responses to retinal antigen by local FoxP3(+) regulatory T cells. Front Immunol.

[61] Hogquist KA, Tomlinson AJ, Kieper WC, McGargill MA, Hart MC, Naylor S, Jameson SC (1997). Identification of a naturally occurring ligand for thymic positive selection. Immunity.

[62] Morris GP, Allen PM (2012). How the TCR balances sensitivity and specificity for the recognition of self and pathogens. Nat Immunol.

[63] Avichezer D, Grajewski RS, Chan CC, Mattapallil MJ, Silver PB, Raber JA, Liou GI, Wiggert B, Lewis GM, Donoso LA, Caspi RR (2003). An immunologically privileged retinal antigen elicits tolerance: major role for central selection mechanisms. J Exp Med.

[64] DeVoss J, Hou Y, Johannes K, Lu W, Liou GI, Rinn J, Chang H, Caspi R, Fong L, Anderson MS (2006). Spontaneous autoimmunity prevented by thymic expression of a single self-antigen. J Exp Med.

[65] Bridges CD, Price J, Landers RA, Fong SL, Liou GI, Hong BS, Tsin AT (1986). Interstitial retinol-binding protein (IRBP) in subretinal fluid. Invest Ophthalmol Vis Sci.

[66] Jeon CJ, Strettoi E, Masland RH (1998). The major cell populations of the mouse retina. J Neurosci.

[67] Panda-Jonas S, Jonas JB, Jakobczyk M, Schneider U (1994). Retinal photoreceptor count, retinal surface area, and optic disc size in normal human eyes. Ophthalmology.

[68] Taylor E, Jennings A (1971). Calculation of total retinal area. Br J Ophthalmol.

[69] Coombes JL, Siddiqui KR, Arancibia-Carcamo CV, Hall J, Sun CM, Belkaid Y, Powrie F (2007). A functionally specialized population of mucosal CD103+ DCs induces Foxp3+ regulatory T cells via a TGF-beta and retinoic acid-dependent mechanism. J Exp Med.

[70] Daniel C, von Boehmer H (2011). Extrathymic generation of regulatory T cells–chances and challenges for prevention of autoimmune disease. Adv Immunol.

[71] Mucida D, Pino-Lagos K, Kim G, Nowak E, Benson MJ, Kronenberg M, Noelle RJ, Cheroutre H (2009). Retinoic acid can directly promote TGF-beta-mediated Foxp3(+) Treg cell conversion of naive T cells. Immunity.

[72] Zhou R, Horai R, Mattapallil MJ, Caspi RR (2011). A new look at immune privilege of the eye: dual role for the vision-related molecule retinoic acid. J Immunol.

[73] Taniguchi RT, DeVoss JJ, Moon JJ, Sidney J, Sette A, Jenkins MK, Anderson MS (2012). Detection of an autoreactive T-cell population within the polyclonal repertoire that undergoes distinct autoimmune regulator (Aire)-mediated selection. Proc Natl Acad Sci U S A.

[74] Cortes LM, Mattapallil MJ, Silver PB, Donoso LA, Liou GI, Zhu W, Chan CC, Caspi RR (2008). Repertoire analysis and new pathogenic epitopes of IRBP in C57BL/6 (H-2b) and B10.RIII (H-2r) mice. Invest Ophthalmol Vis Sci.

[75] Goverman J, Brabb T, Paez A, Harrington C, von Dassow P (1997). Initiation and regulation of CNS autoimmunity. Crit Rev Immunol.

[76] Horai R, Silver PB, Chen J, Agarwal RK, Chong WP, Jittayasothorn Y, Mattapallil MJ, Nguyen S, Natarajan K, Villasmil R, Wang P, Karabekian Z, Lytton SD, Chan CC, Caspi RR (2013). Breakdown of immune privilege and spontaneous autoimmunity in mice expressing a transgenic T cell receptor specific for a retinal autoantigen. J Autoimmun.

[77] Lambe T, Leung JC, Ferry H, Bouriez-Jones T, Makinen K, Crockford TL, Jiang HR, Nickerson JM, Peltonen L, Forrester JV, Cornall RJ (2007). Limited peripheral T cell anergy predisposes to retinal autoimmunity. J Immunol.

[78] Zarate-Blades CR, Horai R, Chen J, Silver PB, Dillenburg-Pilla P, Yamane H, Chan CC, Honda K, Caspi R (2014). Activation of autoreactive T cells by endogenous microflora induces spontaneous autoimmunity in the immunologically privileged retina (BA8P.130). J Immunol.

[79] Caspi RR (2014). Understanding autoimmunity in the eye: from animal models to novel therapies. Discov Med.

[80] Wildner G, Diedrichs-Mohring M (2004). Autoimmune uveitis and antigenic mimicry of environmental antigens. Autoimmun Rev.

[81] Chen J, Qian H, Horai R, Chan CC, Falick Y, Caspi RR (2013). Comparative analysis of induced vs. spontaneous models of autoimmune uveitis targeting the interphotoreceptor retinoid binding protein. PLoS One.

[82] Pauken KE, Linehan JL, Spanier JA, Sahli NL, Kalekar LA, Binstadt BA, Moon JJ, Mueller DL, Jenkins MK, Fife BT (2013). Cutting edge: type 1 diabetes occurs despite robust anergy among endogenous insulin-specific CD4 T cells in NOD mice. J Immunol.

[83] Wells AD (2009). New insights into the molecular basis of T cell anergy: anergy factors, avoidance sensors, and epigenetic imprinting. J Immunol.

[84] Shi G, Lovaas JD, Tan C, Vistica BP, Wawrousek EF, Aziz MK, Rigden RC, Caspi RR, Gery I (2012). Cell-cell interaction with APC, not IL-23, is required for naive CD4 cells to acquire pathogenicity during Th17 lineage commitment. J Immunol.

[85] Forrester JV, Liversidge J, Dua HS (1990). Regulation of the local immune response by retinal cells. Curr Eye Res.

[86] Wang Y, Calder VL, Lightman SL, Greenwood J (1995). Antigen presentation by rat brain and retinal endothelial cells. J Neuroimmunol.

[87] Detrick B, Hooks JJ (2010). Immune regulation in the retina. Immunol Res.

[88] Gregerson DS, Heuss ND, Lew KL, McPherson SW, Ferrington DA (2007). Interaction of retinal pigmented epithelial cells and CD4 T cells leads to T-cell anergy. Invest Ophthalmol Vis Sci.

